# CuSCN as the Back Contact for Efficient ZMO/CdTe Solar Cells

**DOI:** 10.3390/ma13081991

**Published:** 2020-04-24

**Authors:** Deng-Bing Li, Zhaoning Song, Sandip S. Bista, Fadhil K. Alfadhili, Rasha A. Awni, Niraj Shrestha, DeMilt Rhiannon, Adam B. Phillips, Michael J. Heben, Randy J. Ellingson, Feng Yan, Yanfa Yan

**Affiliations:** 1 Department of Physics and Astronomy, and Wright Center for Photovoltaics Innovation and Commercialization (PVIC), University of Toledo, Toledo, OH 43606, USA; dengbing.li@utoledo.edu (D.-B.L.); zhaoning.song@utoledo.edu (Z.S.); sandip.bista@rockets.utoledo.edu (S.S.B.); fadhil.alfadhili@rockets.utoledo.edu (F.K.A.); rasha.awni@rockets.utoledo.edu (R.A.A.); niraj.shrestha@rockets.utoledo.edu (N.S.); adam.phillips@utoledo.edu (A.B.P.); michael.heben@utoledo.edu (M.J.H.); randy.ellingson@utoledo.edu (R.J.E.); 2 Ottawa Hills Junior/senior high school, Ottawa Hills Local Schools, Ottawa Hills, OH 43606, USA; rdemilt20@ohschools.org; 3 Department of Metallurgical and Materials Engineering, The University of Alabama, Tuscaloosa, AL 35487, USA; fyan@eng.ua.edu

**Keywords:** copper thiocyanate, CuSCN, CdTe, zinc magnesium oxide

## Abstract

The replacement of traditional CdS with zinc magnesium oxide (ZMO) has been demonstrated as being helpful to boost power conversion efficiency of cadmium telluride (CdTe) solar cells to over 18%, due to the reduced interface recombination and parasitic light absorption by the buffer layer. However, due to the atmosphere sensitivity of ZMO film, the post treatments of ZMO/CdTe stacks, including CdCl_2_ treatment, back contact deposition, etc., which are critical for high-performance CdTe solar cells became crucial challenges. To realize the full potential of the ZMO buffer layer, plenty of investigations need to be accomplished. Here, copper thiocyanate (CuSCN) is demonstrated to be a suitable back-contact material with multi-advantages for ZMO/CdTe solar cells. Particularly, ammonium hydroxide as the solvent for CuSCN deposition shows no detrimental impact on the ZMO layer during the post heat treatment. The post annealing temperature as well as the thickness of CuSCN films are investigated. Finally, a champion power conversion efficiency of 16.7% is achieved with an open-circuit voltage of 0.857 V, a short-circuit current density of 26.2 mA/cm^2^, and a fill factor of 74.0%.

## 1. Introduction

In the past few years, cadmium telluride (CdTe) solar cells have experienced a rapid performance improvement, with certified record power conversion efficiencies (PCEs) reaching 22.1% for cells and over 18.6% for modules [1,2]. Benefitting from its multi-advantages, including low manufacturing costs, a suitable bandgap, high light absorption coefficients, and, thus, a high theoretical efficiency of over 30% [3], CdTe technology currently dominates the thin-film photovoltaic market. Despite the success in commercialization, it is still challenging to reproduce the record efficiency of CdTe solar cells in research institutes over the world. The efficiency loss is mainly caused by the low p-type conductivity and high electron affinity of CdTe films [4,5], which make them crucial challenges to form a good ohmic contact between CdTe and a metal electrode. The existence of non-ohmic contact produces a back barrier and increases the recombination at the interface of CdTe and the metal electrode, significantly limiting the open-circuit voltage (V_OC_) and fill factor (FF) of CdTe solar cells. To overcome this issue, copper (Cu) is commonly used to improve the conductivity of CdTe and reduce the back-barrier height. However, the concern of long-time stability, due to the ease of Cu migration, inhibits its commercial applications [6]. Group V element (e.g., As and P) doping, as an alternative to Cu doping for the desired long-time stability, has recently attracted intensive investigations. [7,8,9] However, limited by the high capital costs of the equipment used for the in-situ doping and low effective doping level for the ex situ doping (due to aggregations at grain boundaries), group V doping has not been widely used by the researchers [7,10].

Copper thiocyanate (CuSCN), a low-cost and solution-processable material with a high work function of 5.3 eV [11], has been widely used in dye-sensitized and organic-inorganic perovskite solar cells as the hole transport layer to facilitate the carrier extraction [12,13,14]. In 2015, CuSCN was first reported by our group as copper source instead of metallic copper in CdTe solar cells, which delivers an impressive improvement in V_OC_. [11] Recently, CuSCN was successfully used in CdSeTe solar cells, showing an impressive V_OC_ of around 0.860 V and an efficiency of ~17.0%, which makes it a promising back-contact for CdTe solar cells [15]. In this work, the application of solution processed CuSCN as the back contact in pure CdTe solar cells with zinc magnesium oxide (ZMO) as the buffer layer is investigated. ZMO is an emerging buffer layer in CdTe solar cells and helps boost their device performance, due to the reduction in interface recombination at the buffer layer/CdTe interface and parasitic light absorption by the buffer layer [16]. Recently, PCEs of 19.1% and 18.6% for CdSeTe and pure CdTe solar cells, respectively, with ZMO buffer layers have been demonstrated [17]. However, it has also been reported that an oxygen-free atmosphere is required for ZMO/CdTe devices during the CdTe deposition, as well as the post treatments; otherwise, the devices show poor performances, due to the presence of severe S-kinks [18,19]. Additionally, it is reported that the finished ZMO/CdTe devices degrade rapidly when exposing to the ambient atmosphere, likely due to the reaction between water and the ZMO film [20]. All of these suggest that the post treatment of ZMO/CdTe solar cells should be done with great alertness.

Till now, there are only few reports on the optimization of post treatments, especially the back-contact treatment for ZMO/CdTe solar cells. During the optimization processes, we have tried different hole transport materials (e.g., metallic Cu [21], carbon paste [22]) and back surface treatments (e.g., hydroiodic (HI) solution [23], Br methanol solution [24] and MAI solution [25] etching), which have been demonstrated effective in improving the CdS/CdTe device performance. However, most of these processes show adverse effects on the ZMO/CdTe devices, probably caused by the degradation of the ZMO films during the depositions or treatments. In this work, CuSCN dissolved in ammonium hydroxide solution (30 wt.% in water) was used to deposit CuSCN as a back contact for ZMO/CdTe solar cells. ZMO/CdTe devices with metallic Cu doping were used as a reference. Through the optimization of post annealing temperature and the CuSCN film thickness, the solar cells based on the ZMO/CdTe/CuSCN stack show a high PCE of 16.7%, with a V_OC_ of 0.857 V, a short-circuit current density (J_SC_) of 26.2 mA/cm^2^, and a FF of 74.0%. The result demonstrates that CuSCN is a robust hole transport material for CdTe solar cells with ZMO as the buffer layer.

## 2. Materials and Methods

The Schematic illustration of technological steps and investigation design in this work is shown in Figure S1. The devices in this work were fabricated according to our previous report [18]. Fluorine doped tin oxide coated glass (FTO, TEC12; Pilkington NA, Toledo, OH, USA) is used as the substrate after thermal ultrasonic cleaning in Micro-90 detergent (St. Louis, MO, USA) and deionized water at 70 °C. A 80 nm ZMO film was then deposited on the cleaned FTO glass, using a radio frequency sputter system at ambient temperature. The deposition was conducted at 6 mTorr pressure under a mix gas flow of 3% oxygen and 97% helium at a 25 W sputtering power, using a 2-inch ZMO target with 8 wt.% magnesium oxide. Then, a ~3.5 μm CdTe film was deposited in a close-space sublimation (CSS) chamber, with the source temperature of 560 °C, the substrate temperature of 495 °C, and a chamber pressure of 1 Torr. The CdCl_2_ activation treatment was carried out by drop-casting a saturated CdCl_2_ in methanol solution on the CdTe surface, followed with drying naturally and annealing at 420 °C for 20 min at 400 Torr with a 500 sccm helium gas flow. After cooling down, the CdTe film was rinsed by methanol thoroughly to clean the excess CdCl_2_. The CuSCN solution was prepared by dissolving the CuSCN powder in ammonium hydroxide (30 wt.%), or diethyl sulfide with different concentrations (2 mg/mL and 10 mg/mL), and then stirring at room temperature for more than 5 h to facilitate the dissolution. Then, the solution was filtered using a 0.45 μm pore size PTFE filter for the later use. The thickness of CuSCN film was tuned by varying the spin coating speed (2000 rpm and 6000 rpm), and the solution concentration (2 mg/mL and 10 mg/mL). After the CuSCN deposition, the film was heated in our CSS chamber to different temperatures (140 °C, 160 °C, 180 °C) at a ramp rate of ~35 °C/min with a 500 sccm helium flow under ambient pressure. After cooling down naturally, a 40 nm of thick gold was deposited via a shadow mask with an individual area of 0.08 cm^2^ in a thermal evaporator. The optimized reference ZMO/CdTe devices with evaporated Cu metal were fabricated in the same procedure, except evaporating a 3 nm Cu and 40 nm Au bilayer then annealing in ambient pressure at 200 °C for 20 min. The samples for the photoluminescence (PL) measurements were fabricated by depositing 2.5 μm CdTe films followed with the standard CdCl_2_ treatment and the CuSCN or metallic Cu treatment, respectively. All the completed devices described above are listed in Table 1.

The device bandgap diagram simulation was performed using the solar cell capacitance simulator (SACPS) [26], and the parameters were used according to our previous publication [18]. Steady-state photoluminescence (PL) measurement was performed utilizing a 532 nm continuous wave laser at ~5 W·cm^−2^. Samples were excited through the film side. PL signal was detected by a symphony-II Si (CCD) detector (Horiba Scientific, NJ, USA) after a Horiba iHR320 monochromator (Horiba Scientific). The morphological microstructures of the CdTe films were characterized by a nanoscope atomic force microscope (Troy, MI, USA). The solar cell performance was characterized by measuring the current density-voltage (J-V) curves under AM1.5G illumination using a solar simulator (PV Measurements Inc. Point Roberts, WA, USA) and a source meter (Keithley 2400, Beaverton, OR USA). External quantum efficiency (EQE) spectra were performed on a QE system (PV Measurements, Beaverton, OR, USA). Temperature-dependent current-voltage (J-V-T) measurements were performed in a closed-cycle helium cryostat, with a tungsten lamp as a light source, and the temperature was varied from 200 to 310 K, with a step size of 10 K. An inhouse designed LabVIEW (National Instruments, Inc. Beaverton, OR, USA) control program was used to operate the temperature controller, and the Keithley 2400 source-meter for current and voltage data acquisition.

## 3. Results and Discussion

**Figure 1:**
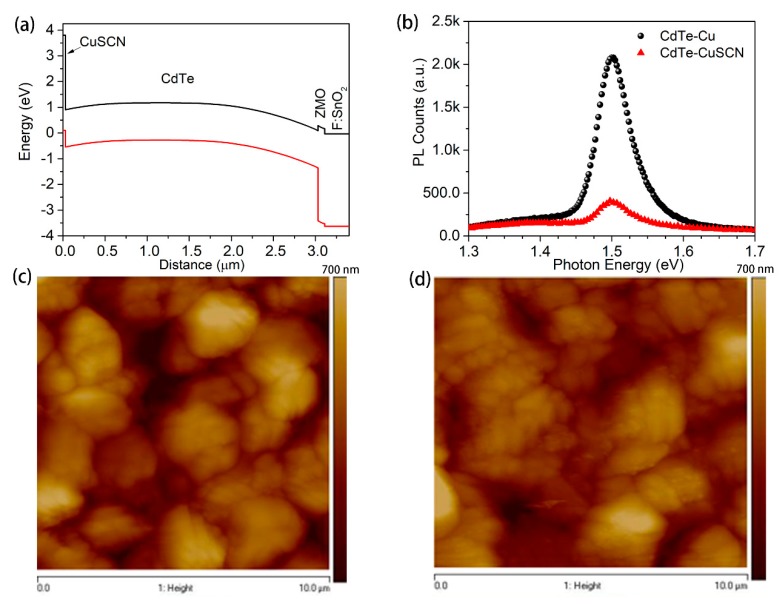
(**a**) Solar cell capacitance simulator (SCAPS) modeling determined band diagram for the device with a structure of fluorine doped tin oxide coated glass (FTO)/zinc magnesium oxide (ZMO)/cadmium telluride (CdTe)/copper thiocyanate (CuSCN)/Au. (**b**) Steady-state PL spectra of CdTe-Cu and CdTe-CuSCN stacks deposited on soda lime glass substrates. Atomic force microscopy images showing the surface morphologies of (**c**) a bare CdTe film and (**d**) a CdTe film deposited with CuSCN.

Figure 1a shows the simulated bandgap diagram of ZMO/CdTe solar cells, with CuSCN as the hole transport layer. The valence band offset between CdTe and CuSCN is favorable for the holes to be extracted from the CdTe layer. Additionally, the low electron affinity with respect to CdTe can favorably repel electrons and prevent them from diffusing into the back electrode. This CdTe/CuSCN heterostructure can eliminate the carrier recombination on the rear side of CdTe, consequently enhancing carrier collection efficiency. The steady-state PL was carried out to further confirm the carrier extraction efficiency (Figure 1b). The CdTe-Cu reference sample exhibits a high intense PL emission peak, centered at 1.50 eV. With the presence of the CuSCN film, the PL emission peak shows pronounced intensity quenching, suggesting strong carrier extraction from the CdTe into the CuSCN. Atomic force microscopy (AFM) measurements were performed to confirm the uniformity of CuSCN film deposited on the CdTe films. The AFM image of bare CdTe film as shown in Figure 1c, exhibits a uniform grain size of ~2 µm, with a root mean squared (RMS) roughness of 147 nm. With the spin-coated CuSCN layer (Figure 1d), the RMS is slightly decreased to 139 nm, indicating a smooth coating of CuSCN particles.

**Figure 2:**
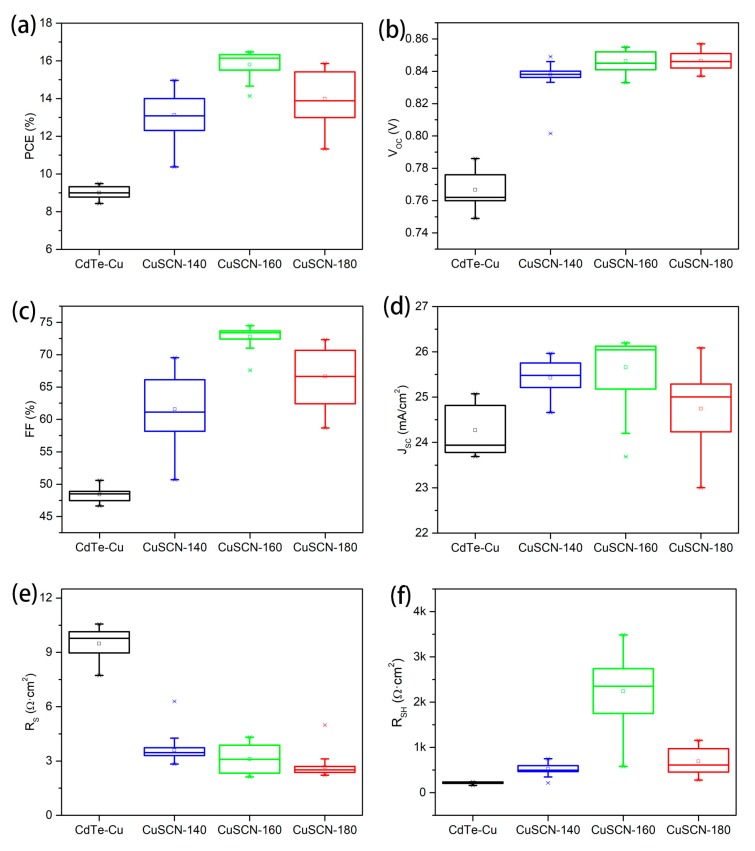
Statistical results for MZO/CdTe solar cell performances of (**a**) PCE, (**b**) V_OC_, (**c**) FF, (**d**) J_SC_, (**e**) series resistance (R_S_), and (**f**) shunt resistance (R_SH_) with metallic Cu and spin-coated CuSCN annealed at different temperatures.

The diffusion of Cu into the CdTe film is a necessary step to improve the ohmic contact between CdTe and the metal electrode. In CdTe/CuSCN stacks, the diffusion of Cu from CuSCN into CdTe not only increases the p-type conductivity of CdTe, but also leaves Cu vacancies in the CuSCN layer, which helps to further increase the work function of CuSCN [27]. To optimize the CuSCN layer, the impact of the annealing temperature of CuSCN was investigated. As shown in Figure 2, the ZMO/CdTe devices with the metallic Cu (named CdTe-Cu hereafter) was used as a control to compare with the ZMO/CdTe devices with a ~30 nm CuSCN layer. The devices with the CuSCN back contact were annealed at different temperatures (140 °C, 160 °C, and 180 °C, named CuSCN-140, CuSCN-160, and CuSCN-180, respectively). With the presence of CuSCN, the devices show overall higher performances than the control devices with the metallic Cu in all the PV parameters. The inferior performance of the reference devices is likely due to the over-diffusion of Cu through CdTe into the ZMO film, which significantly reduces the conductivity of ZMO film by several orders [28,29,30]. We have tried to reduce the annealing temperature and time for the reference devices to reduce the diffusion length of Cu. However, the devices show even worse performances, probably due to the insufficient Cu diffusion. In comparison, CdTe-CuSCN devices annealed at 140 °C for a short duration (a few seconds) outperform the CdTe-Cu devices annealed at 200 °C for 20 min. This result indicates the possibility of reducing the adverse effect caused by the Cu over diffusion through performing Cu annealing at a lower temperature and a shorter holding time. Through lower temperature and shorter time annealing, the Cu diffused into CdTe can be easily confined on the rear side close to the back electrode. The predictable sharp copper concentration profile from the back surface to the bulk not only guarantees a proper Cu concentration on the rear side of CdTe to improve the ohmic contact, but also helps to reduce the Cu related deep trap states in the bulk and front interface caused by excess Cu concentrations [31,32,33]. The proper Cu doping control through our CuSCN treatment can assist to enhance outstanding performance for the CdTe-CuSCN devices.

By increasing the annealing temperature, the mean V_OC_ gradually increases from 0.838 V for CuSCN-140 to 0.847 V for CuSCN-180. The devices annealed at 160 °C deliver the best performance due to the largely improved FF (74.6%), compared to those devices annealed at lower or higher temperatures. This is mainly attributed to the higher hole concentration in CdTe bulk benefited from the proper copper diffusion. Higher hole concentration can boost a higher build-in potential (V_bi_) and lead to less recombination in the depletion region and at the front interface, thereby resulting in larger FF, which can be further confirmed through the improvement of the shunt resistance (R_SH_). Accompanied with the variation of the annealing temperature, the R_SH_ increases from the mean value 490 Ω cm^2^ for CuSCN-140 to 2360 Ω cm^2^ for CuSCN-160. When the annealing temperature is further increased to 180 °C, the mean R_SH_ decreases to 610 Ω cm^2^, due to the excess Cu in the CdTe bulk and at the front interface. As the annealing temperature increases, the series resistance (R_S_) decreases slightly from 3.50 Ω cm^2^ in CuSCN-140 to 3.07 Ω cm^2^ in CuSCN-160, and further to 2.51 Ω cm^2^ in CuSCN-180. The J_SC_ also shows similar trend as the FF and the shunt resistance at different annealing temperatures.

**Figure 3:**
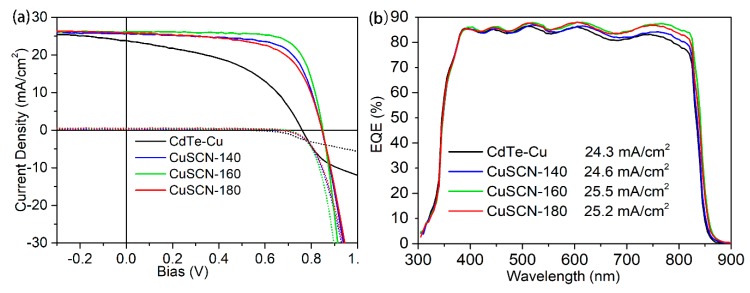
(**a**) Current density-voltage (J-V) and (**b**) external quantum efficiency (EQE) curves for the best devices with different back-contact treatment: evaporated Cu metal and spin-coated CuSCN with different annealing temperatures.

The J-V curves of the best cells of CdTe-Cu and CdTe-CuSCN are plotted in Figure 3a, and more detailed parameters are displayed in Table 2. Comparing to the CdTe-Cu device, the CdTe-CuSCN device shows a higher J_SC_ value, partially due to the reduced recombination at the depletion region and front interface as discussed above. Another reason is the reduced carrier recombination at the back interface between CdTe and the metal electrode, which can be confirmed by the absence of severe roll-over and cross-over effects in the J-V curves at high forward bias for the CdTe-CuSCN devices. The roll-over effect is commonly an indicator of insufficient copper doping on the rear side of CdTe film [34,35,36], and the cross-over effect is due to photoconductivity in the buffer layer which could be caused by the aggregation of copper in the buffer layer. [37] For this sake, the CdTe-Cu device delivers an efficiency of 9.49%, with V_OC_ of 0.786 V, J_SC_ of 24.8 mA/cm^2^, and FF of 48.7%. In contrast, all the CdTe-CuSCN devices show the absence of the roll-over and cross-over effects at forward bias, indicating lower back-barrier heights, and less Cu aggregation at the front interface. Thus, the CdTe-CuSCN devices promise higher FF values (69.1% for the CuSCN-140, 74.0% for the CuSCN-160, and 71.4% for the CuSCN-180). Benefitting from the significantly improved FF, the CuSCN-160 device delivers the best efficiency of 16.5% with V_OC_ of 0.850 V, J_SC_ of 26.2 mA/cm^2^ and FF of 74.0%. External quantum efficiency (EQE) curves were measured to examine the carrier extraction properties for all the devices. As shown in Figure 3b, all the devices show the overlapped curves in short wavelength below 400 nm due to the parasitic absorption of ZMO films. Between 500 to 800 nm, the CdTe-Cu shows the lowest quantum efficiency, compared to the CuSCN ones. The deviation enlarged gradually as the wavelength increases, indicating a larger difference in the recombination properties at the CdTe and metal electrode interface. This can be attributed to two primary reasons: one is that the larger back-barrier height in CdTe-Cu for the hole extraction, and the other one is that the high conduction band offset between CdTe and CuSCN, which can reflect the unfavorable electron diffusion into the CuSCN layer, and thereby suppress the recombination at the back interface. This is consistent with the result of PL quenching for the CdTe/CuSCN film stack. For the CdTe-CuSCN devices annealed at various temperatures, the variation in the quantum efficiency is mainly located at the long wavelengths, probably due to the difference in the Cu concentration at the back surface of CdTe. At higher temperatures, more Cu in the CuSCN layer can diffuse into CdTe. The CuSCN-160 device shows the highest quantum efficiency at long wavelengths, indicating the lowest back barrier height, which can be further confirmed in the temperature dependent J-V measurement, described in the later section. When the annealing temperature is further increased to 180 °C, the Cu concentration at back side is too high, and the interstitial compensation defects Cu_i_, instead of substitutional acceptor defects Cu_Cd_ become dominant, and the back barrier height increases, causing lower quantum efficiencies at long wavelengths for CuSCN-180. Overall, the CuSCN-160 device delivers the highest integrated current density of 25.5 mA/cm^2^, with quantum efficiency over 85% in the whole wavelength range, and the highest value of 88.0% at 605 nm, showing decent carrier transport and extraction properties.

**Figure 4:**
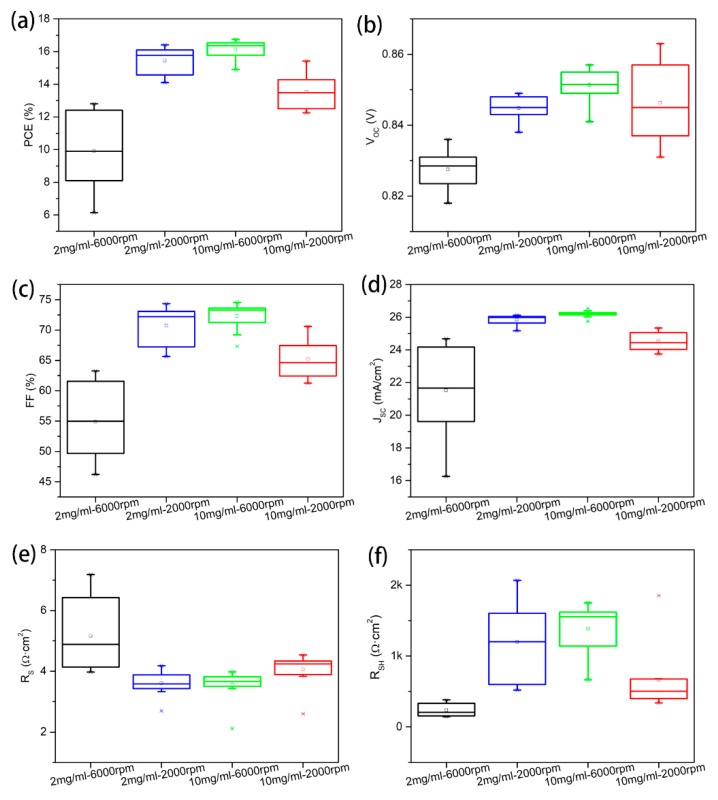
Statistical results for MZO/CdTe solar cell performances of (**a**) PCE, (**b**) V_OC_, (**c**) FF, (**d**) J_SC_, (**e**) series resistance (R_S_), and (**f**) shunt resistance (R_SH_) with different CuSCN film thicknesses tuned by varying the solution concentration and the spin-coating speed.

Due to the high resistivity of CuSCN, the thickness of CuSCN layer is critical for better carrier extraction efficiency. CdTe devices with different CuSCN thicknesses are fabricated using CuSCN solutions with different concentrations (2 and 10 mg/mL) and spin-coating speeds (2000 and 6000 rpm), which are named 2 mg/mL-6000 rpm, 2 mg/mL-2000 rpm, 10 mg/mL-6000 rpm, and 10 mg/mL-2000 rpm, respectively. We tried to measure the CuSCN film thicknesses with different deposition parameters through scanning electron microscopy (SEM) measurements. Unfortunately, the CuSCN films are too thin (less than 30 nm) to be accurately measured through SEM images. The cross-sectional SEM images of the whole devices with the thickest CuSCN film deposited at 2000 rpm using 10 mg/mL CuSCN solution were shown in Figure S2, from which we can know the CuSCN film thickness is about 30 nm, and the others should be thinner than 30 nm. Based on spin-coating features, the thickness of the CuSCN films should rank as 2 mg/mL-6000 rpm < 2 mg/mL-2000 rpm < 10 mg/mL-6000 rpm < 10 mg/mL-2000 rpm, which can be further confirmed by the gradual change of device performance. As shown in Figure 4, the V_OC_ increases gradually as the thickness of CuSCN increases. This can be attributed to the increase of CuSCN film thickness, which supplies more Cu for the diffusion during the annealing treatment, thus, a higher carrier concentration in the CdTe film and higher V_OC_ is generated. The complement of Cu doping concentration also helps to improve all the other J-V parameters, including higher FF, J_SC_, R_SH_, and lower R_S_, yielding a highest efficiency 16.7%, with V_OC_ of 0.857 V, J_SC_ of 26.2 mA/cm^2^, and FF of 74.5%. The detailed information of the best cells with different CuSCN thicknesses are shown in Table 3, and the corresponding J-V curves are shown in Figure S3. As the thickness of CuSCN film increases, PCEs increases from 12.8% to 16.7%. This improvement can be assigned to two major reasons: first, suggested by significant improvement in FF, the CuSCN film became more uniform and the CdTe film can be fully covered by CuSCN film; second, the Cu concentration in CdTe increases gradually due to the increase of CuSCN film thickness, and thus, higher V_OC_ values can be obtained. When the CuSCN film thickness is further increased by spin-coating a 10 mg/mL CuSCN solution at 2000 rpm, V_OC_ is further increased to over 0.863 V, but all the other parameters decrease with a J_SC_ to 25.3 mA/cm^2^, a FF to 70.1%, yielding an efficiency to 15.4%. This is likely ascribed to the high resistance of CuSCN film because, taking an example of the same 10 mg/mL CuSCN solution, decreasing the spin speed from 6000 rpm to 2000 rpm results in increased R_S_ from 3.43 to 4.17 Ω cm^2^.

**Figure 5:**
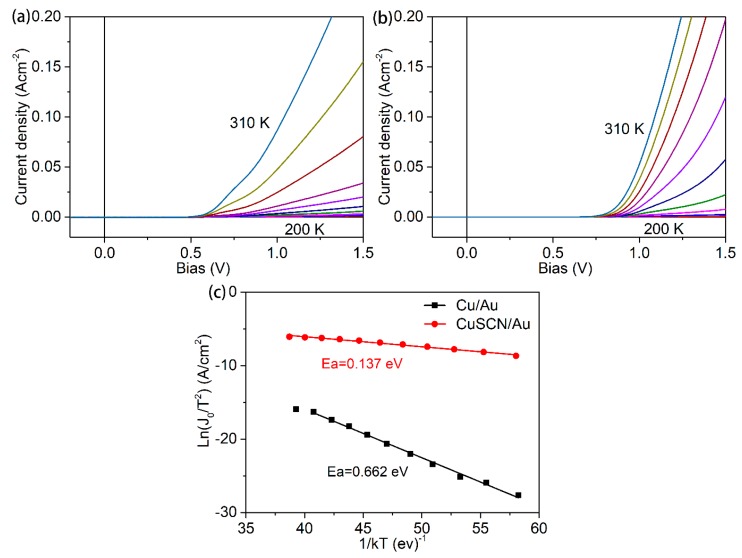
Temperature dependent dark J-V curves for devices with (**a**) Cu metal and (**b**) CuSCN as the back contact. (**c**) Arrhennius plots for the calculation of the back-barrier height for the devices with Cu metal and CuSCN as the back contact.

In order to investigate the origin of device performance improvement with the CuSCN as back contact, the temperature-dependent dark J-V measurements for devices with copper metal (CdTe-Cu) and CuSCN (CuSCN-160) as the back contact were carried out at temperatures ranging from 200 to 310 K, to quantify the back-barrier height as shown in (Figure 5a,b). The temperature dependent dark J-V measurements, as shown in Figure 5a, b, were carried out at temperatures ranging from 200 to 310 K to quantify the back-barrier heights of the devices, with Cu metal and CuSCN as the back contact. For the CdTe-Cu device, the J-V curves show a severe roll-over effect at a high forward bias at room temperature, and the roll-over becomes more pronounced as the temperature decreases to 200 K. In contrast, the CdTe-CuSCN device shows a typical diode behavior, without roll-over in the temperature range of 310 to 250 K. The presence of roll-over effect indicates the existence of a back barrier at the back interface and thereby enhanced carrier recombination. The values of the back-barrier height for the CdTe-Cu and CdTe-CuSCN are calculated according the reference. [38] As shown in Figure 5c, the CdTe-Cu device exhibits a back-barrier height of 0.662 eV, which is extremely high to extract the holes efficiently from the CdTe to the metal electrode. This further explains the origin of low device performances with efficiencies lower than 10%. The CuSCN-160 device possesses a 0.137 eV back-barrier height, which is much lower than that of the CdTe-Cu devices. This small back-barrier height in CuSCN-160 boosts higher carrier extraction efficiency, explaining the origin of the distinctive EQE curves shown in Figure 3b.

Through the careful optimization of the CuSCN treatment, depositing CuSCN film using ammonium hydroxide with water as the solvent was demonstrated to be an efficient means of improving the device performances. However, it has been reported that the performance of ZMO/CdTe device degraded gradually while being exposed to ambient conditions, due to the reaction between water in atmosphere and MgO in ZMO films. [19] To further confirm whether the water in the CuSCN solution will degrade the performance of the ZMO/CdTe solar cells during the annealing treatment, a CuSCN solution without water was used for the CuSCN film deposition. Diethyl sulfide has been widely used as the solvent of CuSCN for hole transport layer deposition. [39,40] For the fabrication of the CdTe-CuSCN device with Diethyl sulfide as solvent, the same procedures used for the CuSCN treatment were used. As shown in Figure S4, the devices using CuSCN in ammonium hydroxide show slightly higher and more uniform performances than those using diethyl sulfide as the solvent. This is because that the uniformity of the CuSCN film deposited using ammonium hydroxide solvent is much better than that using diethyl sulfide as the solvent, which has been confirmed in our previous report [15]. These results further confirm that the performance of our ZMO/CdTe devices is not affected by the water in ammonium hydroxide, demonstrating a robust hole transport layer deposition procedure for ZMO/CdTe solar cells.

## 4. Conclusions

Solution-processed CuSCN treatment with an aqueous solution has been demonstrated to be an efficient procedure for improving the performance of CdTe solar cells with ZMO as the buffer layer. Through the systematic optimization of the CuSCN film thickness and post annealing temperature, the best ZMO/CdTe device achieves an efficiency of 16.7%, with a V_OC_ of 0.857 V, a J_SC_ of 26.2 mA/cm^2^, and an FF of 74.5%. The improvement of the device performance compared with the devices with traditional metallic Cu is mainly attributed to the significantly reduced back-barrier height from 0.662 to 0.137 eV. Our results also indicate that water in ammonium hydroxide solvent has no adverse effect on the ZMO/CdTe devices, indicating a high tolerance of the ZMO/CdTe devices to humidity, and showing a high flexibility for the post treatment of ZMO/CdTe devices in the future.

## Figures and Tables

**Table 1:** Details of all devices fabricated in this work. Note that the buffer layer, CdTe layer, and CdCl_2_ treatment are identical for all the devices as mentioned above.

Devices	Cu Source	Solvent	CuSCN Deposition	Activation Procedure
CdTe-Cu	Cu metal	N/A	TE, 3 nm	200 °C for 20 min
CuSCN-140	CuSCN	NH	SP, 10 mg/mL-6000 rpm	140 °C for 0 min
CuSCN-160	CuSCN	NH	SP, 10 mg/mL-6000 rpm	160 °C for 0 min
CuSCN-180	CuSCN	NH	SP, 10 mg/mL-6000 rpm	180 °C for 0 min
2 mg/mL-6000 rpm	CuSCN	NH	SP, 2 mg/mL-6000 rpm	160 °C for 0 min
2 mg/mL-2000 rpm	CuSCN	NH	SP, 2 mg/mL-2000 rpm	160 °C for 0 min
8 mg/mL-6000 rpm	CuSCN	NH	SP, 10 mg/mL-6000 rpm	160 °C for 0 min
8 mg/mL-2000 rpm	CuSCN	NH	SP, 10 mg/mL-2000 rpm	160 °C for 0 min
diethyl sulfide	CuSCN	DS	SP, 10 mg/mL-2000 rpm	160 °C for 0 min

Note: NH for ammonium hydroxide; DS for diethyl sulfide; TE for thermal evaporation; SP for spin coating.

**Table 2:** Device performance of the best cells in CdTe-Cu and CdTe-CuSCN, annealed at different temperatures.

Samples	V_OC_ (V)	J_SC_ (mA/cm^2^)	FF (%)	Efficiency (%)	R_S_ (Ω cm^2^)	R_SH_ (Ω cm^2^)
CdTe-Cu	0.786	24.8	48.7	9.49	9.78	239
CuSCN-140	0.836	25.9	69.1	15.0	2.95	734
CuSCN-160	0.850	26.2	74.0	16.5	2.70	2580
CuSCN-180	0.851	26.1	71.4	15.9	2.45	1140

**Table 3:** The device performance of the best cells with different CdTe-CuSCN thickness.

CuSCN Deposition	V_OC_ (V)	J_SC_ (mA/cm^2^)	FF (%)	Efficiency (%)	R_S_ (Ω cm^2^)	R_SH_ (Ω cm^2^)
2 mg/mL-6000 rpm	0.830	24.4	63.3	12.8	4.01	381
2 mg/mL-2000 rpm	0.849	26.1	74.1	16.4	3.58	2070
10 mg/ml-6000 rpm	0.857	26.2	74.5	16.7	3.43	1610
10 mg/ml-2000 rpm	0.862	25.3	70.6	15.4	4.17	1350

## Supplementary Materials

The following are available online at https://www.mdpi.com/1996-1944/13/8/1991/s1, Figure S1: Cross-sectional SEM images of complete devices with CuSCN as back contact in different magnification, Figure S2: J-V curves the best cells in devices with different CuSCN thickness, Figure S3: Statistical results for MZO/CdTe solar cell performances of (**a**) PCE, (**b**) V_OC_, (**c**) FF, (d) JSC, (**e**) series resistance (R_S_), and (**f**) shunt resistance (R_SH_) with different CuSCN solutions, Figure S4: Statistical results for MZO/CdTe solar cell performances of (**a**) PCE, (**b**) V_OC_, (**c**) FF, (**d**) JSC, (**e**) series resistance (R_S_), and (**f**) shunt resistance (R_SH_) with different CuSCN solutions.
