# Supplementary material for: CuSCN as the Back Contact for Efficient ZMO/CdTe Solar Cells
Source: Materials (Basel). 2020 Apr 24;13(8):1991. doi: 10.3390/ma13081991 (PMC7215694; doi:10.3390/ma13081991)
Supplement: Supplementary file 1 [file materials-13-01991-s001.pdf]

materials

MDPI

Supplementary Materials

CuSCN as the Back Contact for Efficient ZMO/CdTe Solar Cells

Deng-Bing Li [1], Zhaoning Song [1], Sandip S. Bista [1], Fadhil K. Alfadhili [1], Rasha A. Awni [1], Niraj Shrestha [1], DeMilt Rhiannon [2], Adam B. Phillips [1], Michael J. Heben [1], Randy J. Ellingson [1], Feng Yan [3] and Yanfa Yan [1,*]

[1] Department of Physics and Astronomy, and Wright Center for Photovoltaics Innovation and Commercialization (PVIC), University of Toledo, Toledo, OH 43606, USA; dengbing.li@utoledo.edu (D.-B.L.); zhaoning.song@utoledo.edu (Z.S.); sandip.bista@rockets.utoledo.edu (S.S.B.); fadhil.alfadhili@rockets.utoledo.edu (F.K.A.); rasha.awni@rockets.utoledo.edu (R.A.A.); niraj.shrestha@rockets.utoledo.edu (N.S.); adam.phillips@utoledo.edu (A.B.P.); michael.heben@utoledo.edu (M.J.H.); randy.ellingson@utoledo.edu (R.J.E.)

[2] Ottawa Hills Junior/senior high school, Ottawa Hills Local Schools, Ottawa Hills, OH 43606, USA; rdemilt20@ohschools.org

[3] Department of Metallurgical and Materials Engineering, The University of Alabama, Tuscaloosa, Alabama, 35487, USA; fyan@eng.ua.edu

* Correspondence: yanfa.yan@utoledo.edu

Received: 21 March 2020; Accepted: 21 April 2020; Published: date

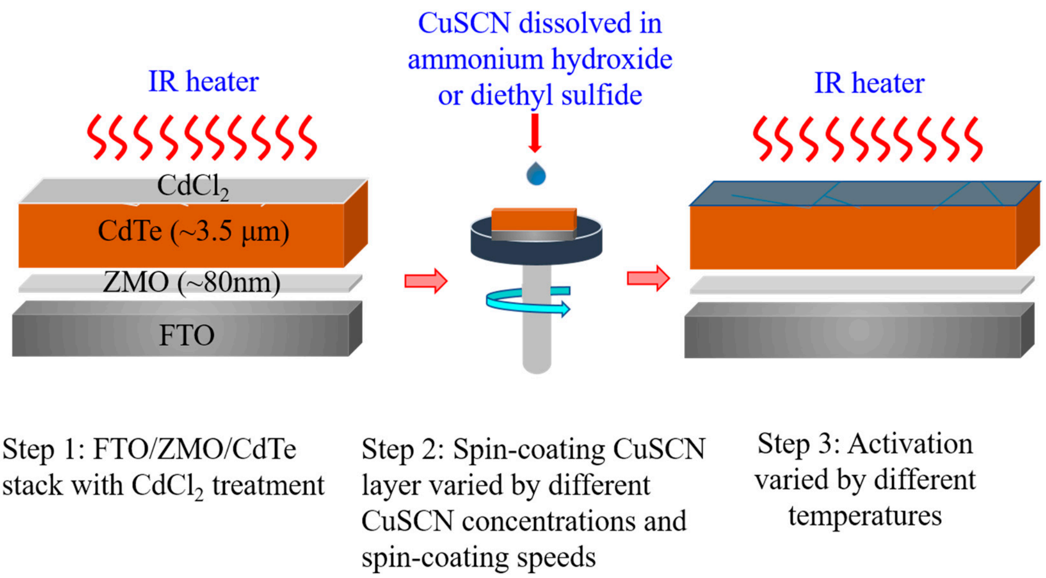

Figure S1. Schematic illustration of technological steps and investigation design in this work.

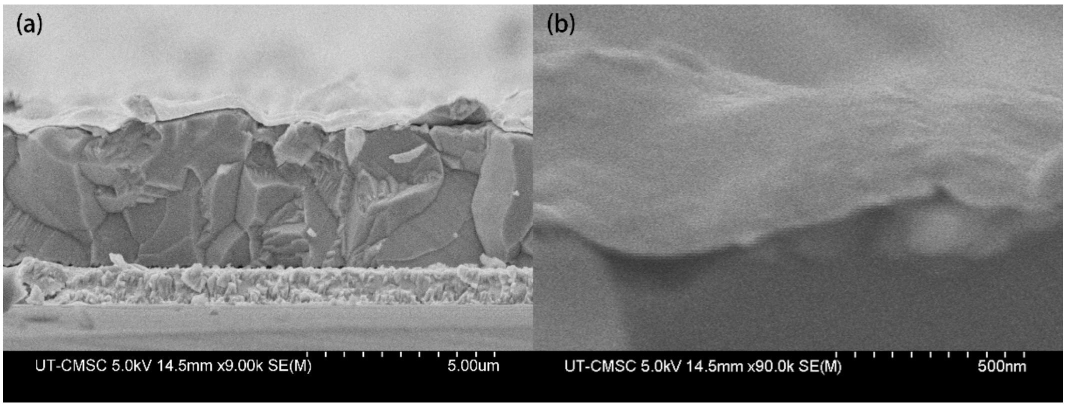

Figure S2. Cross-sectional SEM images of complete devices with CuSCN as back contact in (a) low and (b) high magnification.

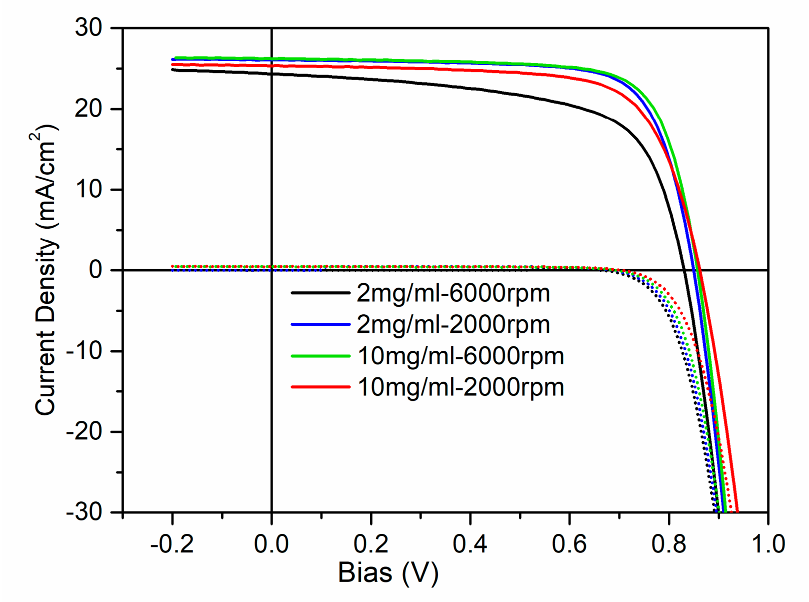

Figure S3. J-V curves the best cells in devices with different CuSCN thickness.

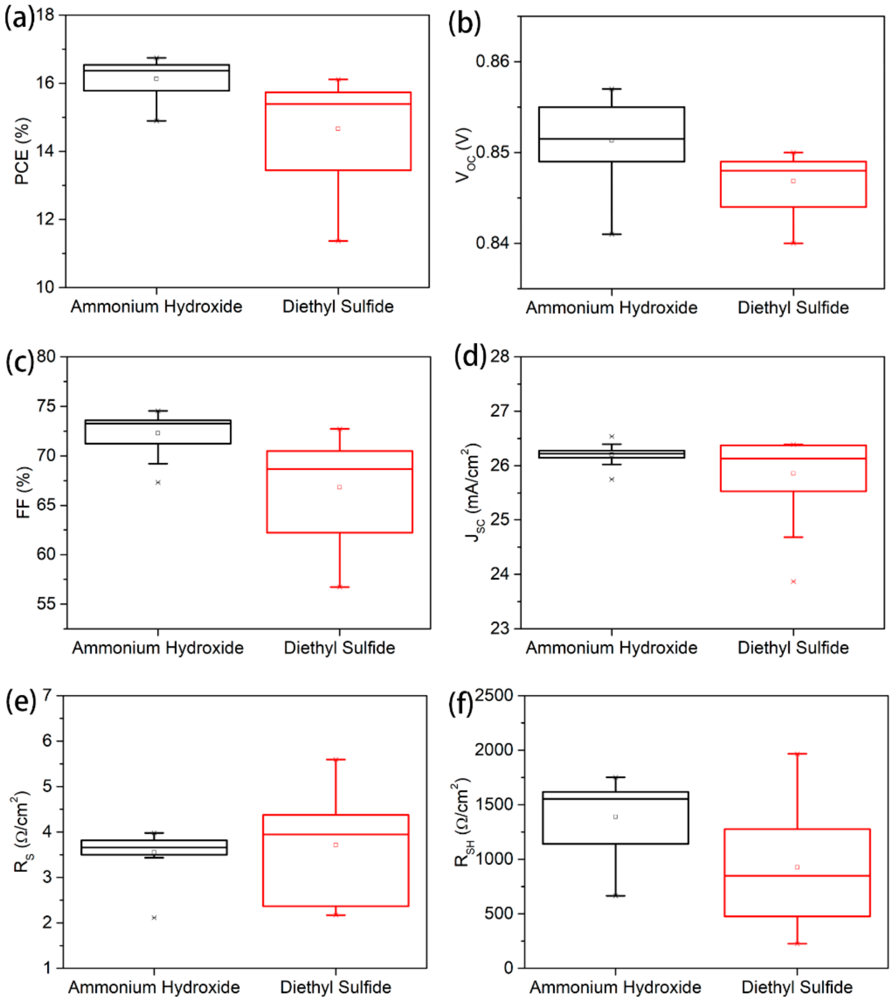

Figure S4. Statistical results for MZO/CdTe solar cell performances of (**a**) PCE, (**b**) V_{OC}, (c) FF, (d) J_{SC}, (e) series resistance (R_S), and (f) shunt resistance (R_{SH}) with different CuSCN solutions.

© 2020 by the authors. Submitted for possible open access publication under the terms and conditions of the Creative Commons Attribution (CC BY) license (http://creativecommons.org/licenses/by/4.0/).
